# Numerical Simulation of Failure Modes of Solid Propellants with Internal Cavities Under Various Loading Conditions

**DOI:** 10.3390/polym18030404

**Published:** 2026-02-04

**Authors:** Kai Liu, Qingchun Yang, Liang Cao, Jianru Wang, Peng Cao

**Affiliations:** 1 National Graduate College for Elite Engineers, Beihang University, Beijing 100191, China; 15509188271@163.com; 2 The Institute of Xi’an Areospace Solid Propusion Technology, Xi’an 710025, China; 3 School of Astronautics, Beihang University, Beijing 100191, China; yangqc@buaa.edu.cn; 4 College of Architecture and Civil Engineering, Beijing University of Technology, Beijing 100124, China; 5 Academy of Aerospace Solid Propulsion Technology, Xi’an 710025, China

**Keywords:** propellant, cavity, impact, GISSMO model, failure mode

## Abstract

The reliability of solid rocket motors depends primarily on the structural integrity of their propellants. Internal cavity defects in the widely used hydroxyl-terminated polybutadiene (HTPB) propellant, formed during manufacturing and service, significantly degrade its mechanical properties and compromise motor safety. This study developed a constitutive model for HTPB propellant based on the generalized incremental stress–strain damage model (GISSMO). The validity of the constitutive model was verified through uniaxial tensile tests conducted at various tensile rates. Based on this constitutive model, numerical simulations were performed to examine the effects of initial modulus, impact rate, and cavity confining pressure on the failure modes of propellants containing cavities with radii from 40 to 100 mm. The results show that the simulation’s force–displacement curve agrees well with the test. The simulation accurately captures the propellant’s transition from elastic–plastic plateau at low rates to elastic response at high rates. The prediction error for the maximum tensile force is less than 5%. For cavities of 80 mm and 100 mm, local stress concentration causes damage to the inner wall, followed by rapid cavity extrusion, collapse, and possible cross-shaped matrix fracture. However, cavities of 40 mm and 60 mm show greater stability, experiencing only volume compression, which rarely causes overall damage. When the propellant’s initial modulus is higher than 24 MPa, damage propagation in large cavities over 80 mm is suppressed. A low modulus worsens structural deformation. At low impact velocity, cavity compression is significant, and the structure remains conformal. At high impact velocity (4000 MPa/s), the cavity stays conformal, the matrix collapses, and the damage value decreases. For 60 mm cavities, damage is localized, and the overall structure is most stable within a confining pressure of 5 to 9.5 MPa. This study clarifies the interaction between engineering parameters and cavity size, providing a basis for optimizing the safety of the propellant structure.

## 1. Introduction

Solid rocket motors are essential in strategic and tactical missiles, launch vehicle boosters, and rapid-response aircraft due to their key advantages: simple structure, high reliability, quick preparation, and long-term storability. Their power source—solid propellant—is a prime example of an energetic composite material. Unlike liquid propellants, solid propellants have a fixed charge structure once manufactured, eliminating the need for complex loading systems and enhancing both rapid response and operational safety. However, solid propellants also present challenges, including relatively low specific impulse, difficulty in precisely regulating thrust and achieving consistent ignition, and significant variations in mechanical properties across a wide temperature range. These issues make maintaining structural integrity and reliability under complex mechanical conditions a major concern in the development and application of solid rocket motors. Particularly under extreme conditions such as high pressure and high-speed impacts, defects within the propellant—such as bubbles (cavities) introduced during preparation and use—can become weak points, leading to mechanical degradation or even structural failure.

Microbubbles are inevitably generated in solid propellants due to the non-uniform mixing of materials in the process of fabrication and service [1], the release of volatile components [2], and the evolution of micro-cracks under thermal stress [3] and other factors. The existence of these bubbles will not only cause non-uniform density of the material, but also become a source of stress concentration, significantly weakening the overall mechanical properties of the propellant [4,5]. Especially in high pressure and high-speed impact environment [6,7], bubbles can significantly change the deformation and failure characteristics of propellant. Therefore, the quantitative study of the effect of bubble size on the mechanical properties of propellant materials is the key link to understand its dynamic failure behavior and improve the reliability and safety of propellant. At the same time, it also provides theoretical support for optimizing the propellant forming process.

A large number of studies have been conducted on the evolutionary behavior of bubbles in fluid and viscoplastic media, mainly focusing on the compression deformation, collapse and compression fracture mechanism of bubbles under external pressure [8,9]. High-impact microjets and shock waves are generated by bubble collapse under impact loading in liquids [10,11]. However, studies on viscoplastic media have shown that bubbles undergo creep deformation and eventually rupture under loading [12,13,14]. In addition, VialI and Narchi [15] investigated the effect of bubble size on the viscosity and stability of foam fluids and found that the upper limit of the zero-shear plateaux region decreases as the bubble size decreases. Qin et al. [16] investigated the dynamics of lipid-coated microbubbles in viscoelastic media, showing that bubble size (initial radius), compression (asymmetric dynamic behavior), and rupture (lipid shell buckling) all affect both the microbubbles and the surrounding medium. Moschopoulos et al. [17] the motion of a knife-edge-shaped bubble in a viscoelastic material is investigated. Recently, Li et al. [18] the rising behavior of bubbles in gelled propellants is studied. It is found that the bubble pairs condense and rise with the increase in bubble diameter. Moreover, under high liquid viscosity, even if the surface tension is greatly reduced, the vortex inside the bubble is difficult to break through the bubble boundary [19]. Dong et al. [20] in situ observation of the bubble evolution in the propellant at different tensile strain rates shows that the bubble structure shows obvious rate-dependent characteristics. Although these studies provide an important reference for understanding the behavior of bubbles in complex materials, there is still a lack of quantitative research on the effect of bubble size on the shock compression performance of solid propellants at home and abroad.

In terms of solid propellant constitutive model research, existing studies mainly focus on the overall mechanical response under tensile or compressive conditions, especially the constitutive behavior at different tensile rates [21,22,23]. Moreover, some scholars have also incorporated the porosity into the damage constitutive model of propellant to simulate the mechanical response of propellant [24,25]. However, most of these models are based on the homogeneous material assumption [26,27] and do not fully consider the effect of bubble size on the dynamic mechanical behavior of propellants. In contrast, the generalized incremental stress–strain damage model (GISSMO) constitutive model can effectively describe the nonlinear behavior of materials at high strain rates [28,29,30], and particularly excelling in considering microstructure factors [31,32]. By introducing damage evolution variables and failure criteria into the framework of solid mechanics, GISSMO can simulate the entire process of crack initiation, growth and coalescence caused by voids or bubbles in materials [33]. Moreover, the decay exponential function can control the rate of energy dissipation for a given stress decay state [34]. Mao et al. [35] based on the force–displacement curve from tensile test, the attenuation index and mesh regularization function of GISSMO were calibrated, and the ballistic response of Armox 500T steel under dynamic impact was simulated. Rickhey et al. [36] studied the damage and fracture behaviors of thermoplastic materials at high strain rates using GISSMO. It is found that the damage and fracture parameters can accurately predict the damage and fracture under various conditions. Cui et al. [37] based on GISSMO, the ductile fracture behavior of viscoelastic–plastic materials is simulated and the sensitivity of structural defects is evaluated. In summary, the GISSMO model has significant advantages in capturing the strain rate effect, stress state dependence and damage evolution of materials, which provides a potential modeling method for simulating complex failure modes under the introduction of bubbles. However, the GISSMO constitutive model has not been used to simulate the failure mode of solid propellant containing cavities under dynamic impact. Therefore, it is of great significance to develop the failure simulation method of propellant based on GISSMO constitutive model for understanding the influence of cavity size on the mechanical properties of propellant.

To address the identified research gaps, this study developed a numerical simulation framework based on the GISSMO constitutive model to quantitatively examine how cavity size influences the dynamic compression failure behavior of solid propellants. First, a GISSMO model tailored to solid propellants was constructed and calibrated. Next, uniaxial tensile experiments at varying strain rates were performed to collect mechanical response data, and the model’s accuracy was validated through finite element inverse analysis. Systematic simulations were then conducted to explore the damage evolution and failure processes in propellant models with cavities of different radii under dynamic compression. Finally, using the validated model, the impact of key parameters—such as initial modulus, impact rate, and cavity confining pressure—on the failure modes of propellants with varying cavity sizes was investigated. This research provides new analytical tools and insights into the damage mechanisms of cavities in solid propellants, offering valuable theoretical and engineering guidance for structural safety assessments and enhancing propellant service reliability.

## 2. Materials and Methods

### 2.1. Raw Materials

The propellant used was of the HTPB type. It used glycidyl azide polymer crosslinked with straight-chain terminal hydroxyl groups as the binder, triethyleneglycol dinitrate as the energetic plasticizer, and ammonium perchlorate combined with nitramine particles as the oxidizer and high-energy filler. It also contained small amounts of bonding agents, curing catalysts, and stabilizers. All raw materials were vacuum-dried before use to remove moisture. Test specimens were precision-molded, cured, and then CNC-machined to ensure dimensional consistency. After machining, the critical dimensions of each specimen were measured three times using vernier calipers (with 0.02 mm accuracy), and the average value was recorded. Deviations between the actual and nominal dimensions were maintained within ±0.1 mm.

### 2.2. Experimental Testing

**Figure 1:**
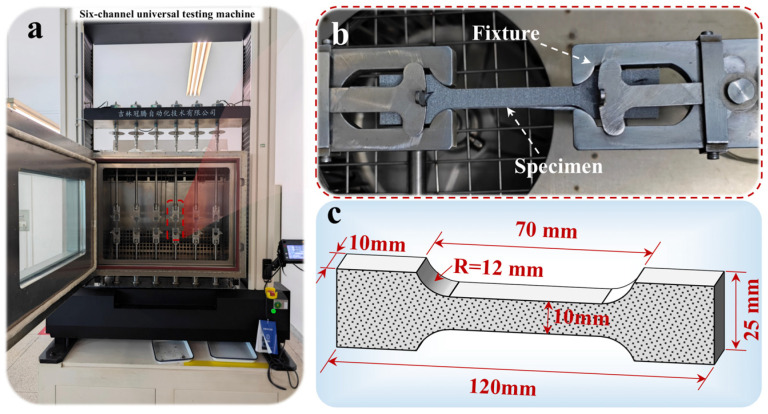
Testing equipment and specimen dimensions. (**a**) Six-channel uniaxial tensile testing machine, (**b**) Specimen testing fixtures and mounting configuration, (**c**) Specimen geometric dimensions.

Tensile tests were performed using a six-channel universal testing machine (Instron 5967, Figure 1a). The machine has a maximum test force of 50 kN, a displacement speed range of 0.001–5000 mm/min, a strain resolution of 0.001 mm, and a load resolution of 1 N. Specimens were secured using pneumatic push-out grips (Figure 1b) with a clamping pressure of 0.6 MPa. Sandpaper was placed between the specimen and the grip contact surfaces to prevent slippage. The dimensions of the propellant specimen are shown in Figure 1c. During testing, force signals were directly acquired from the machine’s built-in sensors, and displacement signals were recorded using the crosshead displacement system. Data acquisition was set to 100 Hz to ensure complete capture of the failure process. To characterize the strain rate sensitivity of the propellant under quasi-static to dynamic conditions, four orders of magnitude of constant crosshead displacement rates were selected: 2, 20, 200, and 2000 mm/min. These rates correspond to nominal initial strain rates in the gauge section of approximately 0.00067, 0.0067, 0.067, and 0.67 s^−1^. Raw force–displacement data were converted into engineering stress–strain curves using the initial geometric dimensions of the specimen’s gauge section. At least six valid tests were performed for each set of test conditions (each strain rate). To exclude outliers, Raita’s criterion was applied to screen the fracture strength and fracture elongation data. The final data used for constitutive model calibration were the arithmetic mean of valid test results for each parameter.

## 3. Finite Element Model Development

### 3.1. Propellant Uniaxial Tensile Model

**Figure 2:**
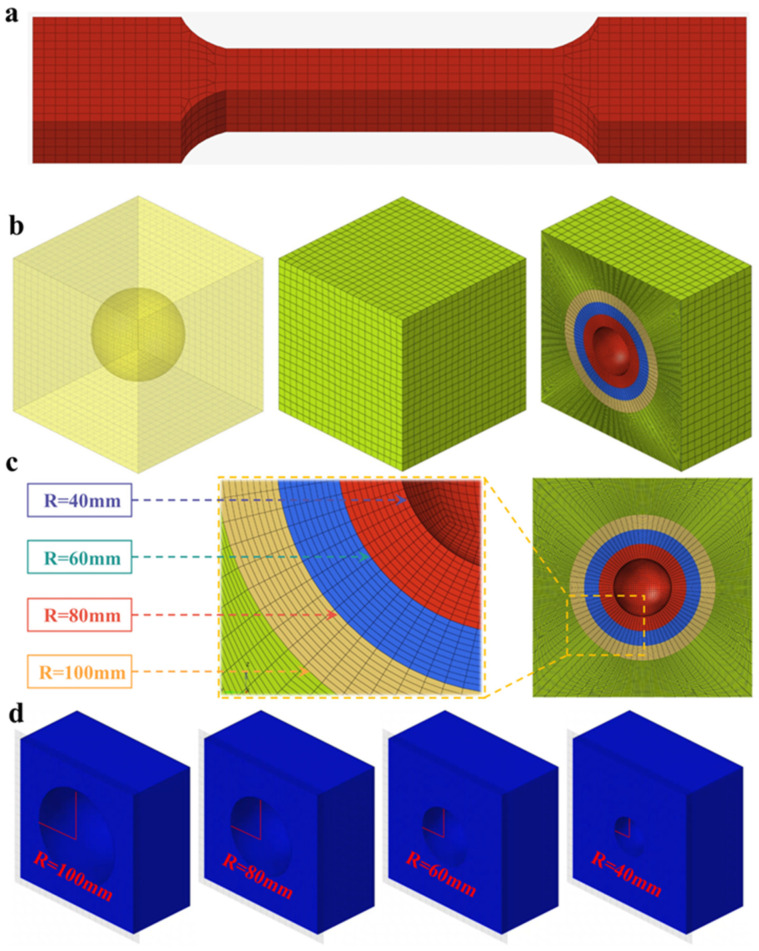
Uniaxial tensile specimen model and propellant finite element model with cavities of different radius: (**a**) uniaxial tensile specimen model, (**b**) model of the propellant containing cavities, (**c**) grid details of cavities of different radius, and (**d**) models with cavities of different radius.

The numerical simulations were conducted using the explicit finite element code LS-DYNA (Version R11.1, Livermore Software Technology Corporation, Livermore, CA, USA) [38] to verify the experimental data and the true stress–plastic strain curve obtained through fitting. The finite element simulation specimen was modeled to match the size of the actual specimen (Figure 2a). The *MAT_PIECEWISE_LINEAR_PLASTICITY material card in LS-DYNA 24 was used to define the mechanical properties of the propellant material, while the *DEFINE_CURVE keyword was employed to input the flow stress–plastic strain curve.

### 3.2. Propellant Model with Cavities

In real engines, the overall dimensions of the propellant charge are determined by design, while defects (such as bubbles) introduced during manufacturing or service are treated as independent variables. Furthermore, when external constraints are fixed, larger defects can cause more significant local stress concentrations and lead to earlier damage initiation. Therefore, this study aimed to simulate this specific operating condition and investigate how the size of individual bubble defects affected the dynamic failure behavior within a segment of a solid rocket motor propellant charge with fixed geometric boundaries. Moreover, this study considered the extreme scenario in which macroscopic defects might form in solid propellants during manufacturing or service due to process anomalies, material degradation, or mechanical damage. It used a cavity model with diameters ranging from 40 to 100 mm. The goal is to quantitatively assess the impact of these critical defects on the material’s dynamic failure behavior.

In this study, a cubic specimen measuring 300 mm × 300 mm × 300 mm was first created, with spherical cavities of 40 mm, 60 mm, 80 mm, and 100 mm in diameter excavated within it (Figure 2b,c). The geometric model was then subjected to multiple cuts to generate a complete hexahedral mesh. Lastly, an increasing pressure load of 8 MPa was applied to all six external faces of the structure. Under this simulated condition, larger cavity sizes led to their boundaries being closer to the loading surface. This shows that the absolute size effect of defects is coupled with the “defect-boundary relative distance” effect.

Additionally, this study focused on the dominant mechanisms of stress concentration, damage initiation, and propagation triggered by the solid matrix as a geometric defect within the cavity. Therefore, the cavity in the model was simplified as a vacuum cavity, disregarding the dynamic behavior of the internal gas. This simplification may have introduced deviations when the cavity scale was large or the loading rate was relatively low, indicating an area for future refinement.

## 4. Damage Definition

GISSMO, integrated into LS-DYNA, describes fracture behavior in solid propellant with cavities. It predicts impact failure under different strain rates and stress states. The GISSMO failure model combines the constitutive model with *MAT_ADD_EROSION to define material failure. This study used the *Mat_024 model, its mechanical property curve was obtained earlier. The GISSMO algorithm governs the evolution of the damage factor (*D*) over time, which is represented by the exponential equivalent plastic strain formula provided in Equation (1) [35].

(1) $$D={\left(\frac{\varepsilon^{pl}}{\varepsilon_{f}\left(\eta ,\bar{\theta }\right)}\right)}^{n}$$

where *n* is the damage index that defines the nonlinear damage evolution, with a value of 1.5. $\varepsilon^{pl}$ is the current equivalent plastic strain and $\varepsilon_{f}\left(\eta ,\bar{\theta }\right)$ is the function of the equivalent plastic strain at fracture with stress triaxiality (*η*) and Lode angle parameter ($\bar{\theta }$). When *D* = 0, the material is intact. But when *D* = 1, the material fails. *η* defines the ratio of hydrostatic stress (*σ*_*m*_) or hydrostatic pressure (*p*) to von Mises stress (*σ_v_*_*m*_) [39]. Lode angle parameter ($\bar{\theta }$) is defined as the third deviatoric stress invariant to quantify the stress departure state. Defined by Equations (2) and (3) [40]:

(2) $$\eta =\frac{\sigma_{m}}{\sigma_{vm}}=-\frac{p}{\sigma_{vm}}$$

(3) $$\bar{\theta }=cos\left(3\theta \right)=\frac{27}{2}\frac{det\left(s\right)}{\sigma_{vm}^{3}}$$

here $\sigma_{m}=\frac{\sigma_{1}+\sigma_{2}+\sigma_{3}}{3}$, $\sigma_{\upsilon m}=\sqrt{\frac{{\left(\sigma_{1}-\sigma_{2}\right)}^{2}+{\left(\sigma_{2}-\sigma_{3}\right)}^{2}+{\left(\sigma_{3}-\sigma_{1}\right)}^{2}}{2}}$, where *σ*_1_, *σ*_2_, *σ*_3_ are the three principal stresses, *s* is the deviatoric stress tensor, and *θ* is the Lode angle.

In the GISSMO algorithm, under non-proportional loading, the damage factor (*D*) measures material degradation, and the instability factor (*F*) measures material instability. Specifically, *F* quantifies the tendency for necking to develop under dynamic loading. *F* ranges from 0 to 1 and serves as an indicator of the dynamic transition from uniform plastic deformation to localized necking. Both factors can be tracked over time [35].

(4) $$\Delta D=\frac{n}{\varepsilon_{f}\left(\eta ,\bar{\theta }\right)}D^{\left(1-\frac{1}{n}\right)}\Delta \varepsilon^{pl}$$

(5) $$\Delta F=\frac{n}{\varepsilon_{crit}\left(\eta ,\bar{\theta }\right)}F^{\left(1-\frac{1}{n}\right)}\Delta \varepsilon^{pl}$$

Equations (4) and (5) define incremental damage and instability as functions of the current damage factor and instability measure. The critical strain $\varepsilon_{crit}\left(\eta ,\bar{\theta }\right)$, or equivalent plastic strain at necking onset, serves as a weighting function for the actual stress state and the incremental equivalent plastic strain. This point is often defined by the instability factor, which indicates the onset of diffuse necking. At this stage, accumulated damage couples with the stress tensor, enabling material softening in line with Lemaitre’s classical effective stress principle [41].

(6) $$\sigma_{m}^{*}=\sigma_{c}\left(1-D^{*}\right)$$

(7) $$D^{*}=\left\{\begin{array}{cc} 0 & \mathrm{if}\;F<1\\ {\left(\frac{D-D_{crit}}{1-D_{crit}}\right)}^{m} & \mathrm{if}\;F=1 \end{array}\right.$$

Among them, $\sigma_{m}^{*}$ is the modified stress of damage coupling, $\sigma_{c}$ is the current stress, and $D_{crit}$ is the critical threshold damage value corresponding to *F* = 1. The modified stress can be calculated as [35]:

(8) $$\sigma^{*}=\sigma_{c}\left(1-{\left(\frac{D-D_{crit}}{1-D_{crit}}\right)}^{m}\right)\;\mathrm{for}\;D\geq D_{crit}$$

where *m* is the attenuation index, which can be defined as a function of mesh size to control stress attenuation and energy dissipation, with a value of 1.5.

## 5. Results

### 5.1. Comparison of Uniaxial Tensile Test and Simulation Results

**Figure 3:**
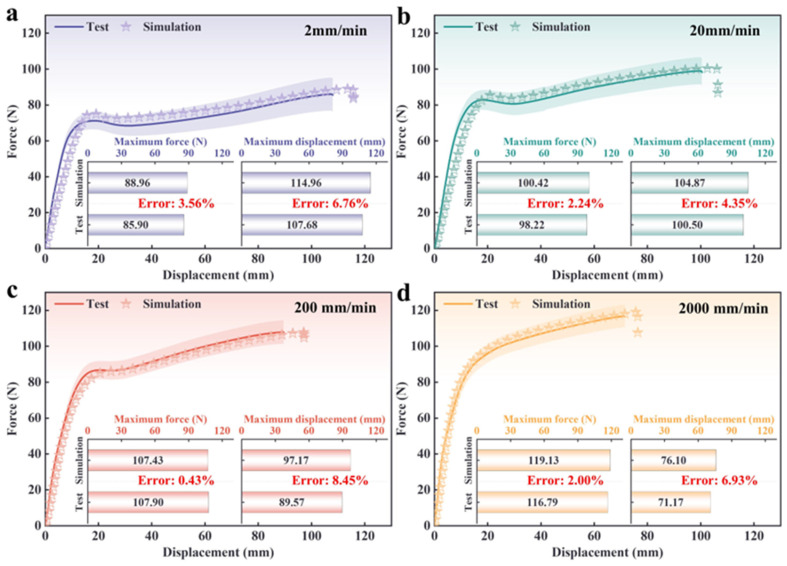
Comparison of test and simulation results of propellants at different tensile rates: (**a**) 2 mm/min, (**b**) 20 mm/min, (**c**) 200 mm/min, (**d**) 2000 mm/min.

Figure 3 presents data from uniaxial tensile tests and numerical simulations of solid propellant specimens at four tensile rates: 2 mm/min, 20 mm/min, 200 mm/min, and 2000 mm/min. At a tensile rate of 2 mm/min (Figure 3a), the curves for both materials show consistent trends, including a gradual increase during the elastic stage, sustained deformation during the plastic stage, and a subsequent flattening of the force response. The simulation’s maximum force error is 3.56%, and its displacement error is 6.76%, confirming predictive precision at low rates. At 20 mm/min (Figure 3b), the experimental and simulated curves nearly coincide. The elastic region ascends more sharply, and the post-peak response forms a narrower plateau. Simulation errors for maximum force and displacement are 2.24% and 4.35%, respectively, indicating strong agreement. At a tensile rate of 200 mm/min (Figure 3c), the experimental and simulated curves coincide completely. The force rises abruptly from loading to peak, with minimal plastic transition, illustrating a distinctly elastic response. Maximum force error is negligible (0.43%), while displacement error increases (8.45%), but primary mechanics remain accurate. At 2000 mm/min (Figure 3d), the experiment and simulation exhibit similar patterns, showing a swift, continuous rise without a marked plateau, which reflects the high-speed dynamic behavior. The maximum force error (2.00%) is low, and the displacement error (6.93%) matches lower rates.

A comparative analysis of the results at four tensile rates shows that the experimental and simulated curves gradually converge from 2 mm/min to 200 mm/min, with the greatest overlap at 200 mm/min. At 2000 mm/min, a slight divergence appears, possibly due to rate-dependent constitutive effects at higher strains. As the tensile rate increases, both maximum forces rise while displacement capacity drops, paralleling lower deformation at higher speeds. Errors for force and displacement remain low, confirming the simulation’s fidelity in capturing rate hardening and deformation trends. Maximum force simulation errors (0.43–3.56%) and moderate displacement errors emphasize the model’s predictive power for mechanical limits.

Although simulation accuracy remains high across all stretching rates, specific limitations of the model are evident. While maximum force errors are consistently low, displacement errors fluctuate, exposing an inadequate representation of rate-dependent damage mechanisms in the simulation. Notably, the simulation does not account for dynamic particle debonding and matrix cracking that occur during large deformations [42,43,44], resulting in poorer agreement at 2000 mm/min compared to 200 mm/min. These gaps indicate the model’s limited capacity to capture dynamic responses at very high rates. Future refinement of the constitutive model, along with the inclusion of high-speed meso-damage experiments, could address these deficiencies and enhance simulation accuracy.

### 5.2. Damage Evolution of Propellant with Different Cavity Radius

**Figure 4:**
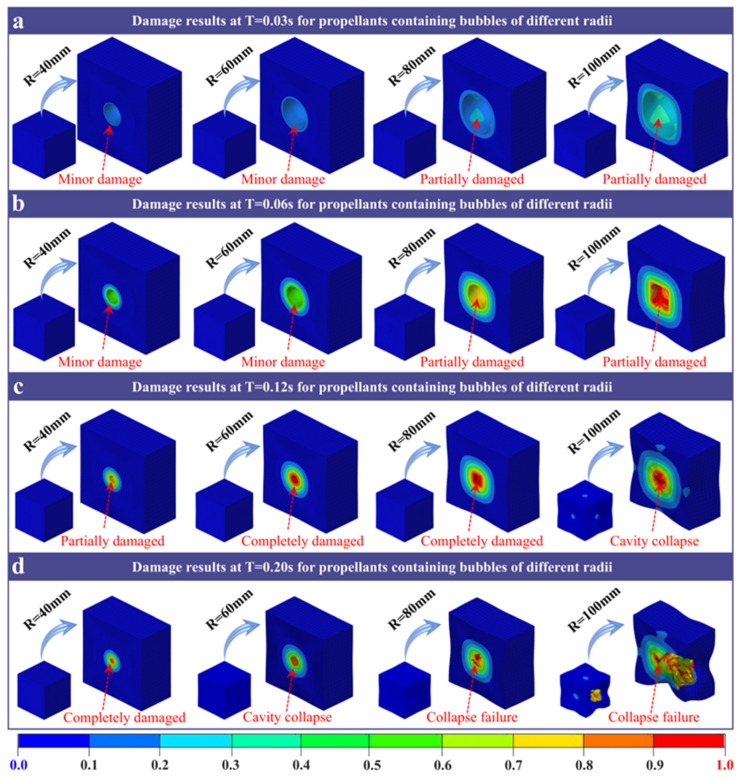
Damage nephograms of propellants with cavities of different radius under different loading times: (**a**) T = 0.03 s, (**b**) T = 0.06 s, (**c**) T = 0.12 s, (**d**) T = 0.20 s.

Figure 4 shows the damage evolution of propellants with different cavity radii over time. At 0.03 s (Figure 4a), samples with cavity radius of R = 40 mm and R = 60 mm show only small, uniform damage areas on the inner wall. In contrast, samples with cavity radii of R = 80 mm and R = 100 mm begin to exhibit local damage in the inner wall and surrounding matrix. This is accompanied by a decrease in cavity compression. At 0.06 s (Figure 4b), the specimen with an R = 100 mm cavity radius approaches the critical point of failure. Its cavity section becomes square, the volume is maximally compressed, and the sample undergoes collapse with local matrix damage. The R = 80 mm, R = 60 mm, and R = 40 mm samples retain circular cavity cross-sections. They exhibit only minor damage to the inner walls. Notably, all cavity radii are reduced at this moment due to external pressure. At 0.12 s (Figure 4c), specimens with R = 40 mm and R = 60 mm cavity radius continue to have circular cross-sections. The R = 60 mm sample’s inner wall partly reaches the damage limit, while the R = 40 mm sample does not. The R = 80 mm sample now has a square-shaped cavity cross-section, and its inner wall is fully damaged. The R = 100 mm specimen sustains typical crushing failure with a four-leaf clover failure mode. The matrix’s damaged area extends outward in a cross shape. By 0.20 s (Figure 4d), a complete collapse occurs in the R = 100 mm cavity specimen. Gas is expelled through the cross-damage regions, resulting in significant damage to the matrix. The R = 80 mm and R = 60 mm samples exhibit central region collapse. However, due to the matrix material coating, the overall samples show only slight collapse. The R = 40 mm sample still retains a circular cavity cross-section, with only partial inner wall damage at the limit.

Overall, as the load acts continuously, damage spreads from the inner wall outward into the propellant matrix. Before the cavity bursts, the cavity’s cross-section becomes square, like the matrix. This indicates higher pressure on the sides of the cavity. Larger cavity radius (R = 80 mm and R = 100 mm) first exhibit severe inner wall damage at the same load time. These cavities rapidly approach failure because they experience higher inner wall pressure. Continued external loading causes the inner wall material to crack and propagate micro-cracks. This leads to local fracture and, eventually, large-scale material damage as the cavity’s inner wall collapses locally. In contrast, smaller radius samples (R = 40 mm and R = 60 mm) benefit from the matrix’s buffer. They have less inner wall damage and a slower spread across regions. Under the same load, small cavities primarily exhibit compression, rather than rupture or collapse. Therefore, future propellant designs should consider the effects of cavity size on damage and failure to maintain material reliability.

### 5.3. Analysis of Propellant Damage Patterns Under Different Conditions

The propellant will experience the change of initial modulus, impact velocity, and cavity confining pressure in actual service [45,46]. For this purpose, the damage modes of propellants under four initial moduli *E* (8, 16, 24, and 32 MPa), four impact rates *V* (4, 40, 400, and 4000 MPa/s), and four cavity confining pressures *P* (5, 6.5, 8, and 9.5 MPa) are analyzed in this section.

#### 5.3.1. Effect of Propellant Initial Modulus on Failure Mode

**Figure 5:**
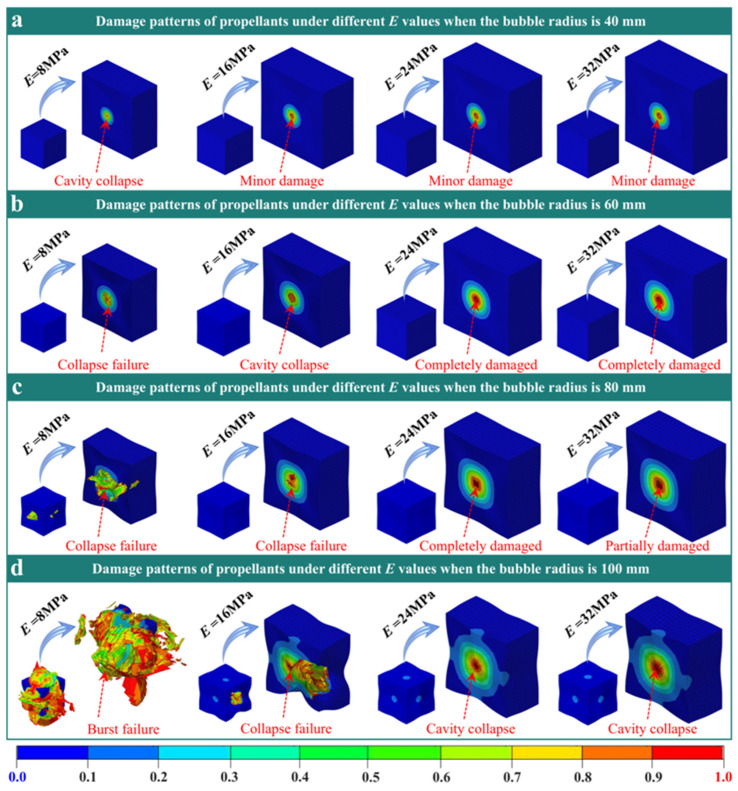
Damage nephogram of propellant containing cavities with different radius at different *E*. (**a**) R = 40 mm, (**b**) R = 60 mm, (**c**) R = 80 mm, (**d**) R = 100 mm.

The final damage mode of the propellant containing four cavities’ radii at different initial moduli *E* is presented in Figure 5. Here, the loading rate *V* and cavity confining pressure *P* are both 40 MPa/s and 8 MPa. For the propellant with a cavity radius of R = 40 mm (Figure 5a), significant overall structure deformation occurs when *E* = 8 MPa due to the low modulus; cavities in the cross-section collapse completely. When *E* ≥ 16 MPa, deformation is minimal. In these higher modulus cases, the cavities retain their circular shape, with damage manifesting as a local ring centered on the cavity. A cavity radius of R = 60 mm (Figure 5b) leads to more pronounced structural deformation at *E* = 8 and *E* = 16 MPa because of the combination of low modulus and larger cavity size. In these cases, the entire cavity section collapses, and the affected area exceeds that of the 40 mm cavity scenario. With *E* = 24 MPa and *E* = 32 MPa, structural deformation lessens, cavities return to a circular form, and damage remains confined to a ring around them. At a cavity radius of R = 80 mm (Figure 5c), further reduction in deformation is observed for the *E* = 8 MPa propellant. However, the cavities are squeezed out, disrupting the structural integrity. For *E* = 16 MPa, the structure remains intact, but cavities collapse fully. However, propellants at *E* = 24 MPa and *E* = 32 MPa avoid collapse; instead, the cavity cross-section becomes square. Lastly, for the 100 mm cavity radius (Figure 5d), both *E* = 8 MPa and *E* = 16 MPa result in clear structural crushing, whereas at *E* = 24 MPa and *E* = 32 MPa, the cavities collapse, although the overall structure is preserved. These results demonstrate that a lower modulus favors cavity collapse and deformation, whereas a higher modulus maintains the cavity shape and limits structural change.

The physical mechanism is tied to modulus. Propellant with a low modulus deforms readily, making stress at cavity defects transfer outward more easily as large deformations occur. This situation causes cavity collapse, shrinkage, and widespread damage. In contrast, propellants with high modulus are stiffer, limiting deformation and keeping stress concentrated near the cavity. As a result, cavities maintain their shape and damage appears locally. Larger cavity radius expands the range of stress concentration and worsens damage, especially in low-modulus materials. From an engineering perspective, matching the initial modulus with cavity size ensures stability. For small cavities (R = 40 mm), *E* should remain at or above 16 MPa to prevent collapse. For medium and large cavities (R = 60–100 mm), *E* needs to be at or above 24 MPa to limit propagation and damage. Controlling cavity defect size further reduces the risk of failure.

#### 5.3.2. Effect of Impact Velocity on Failure Mode

**Figure 6:**
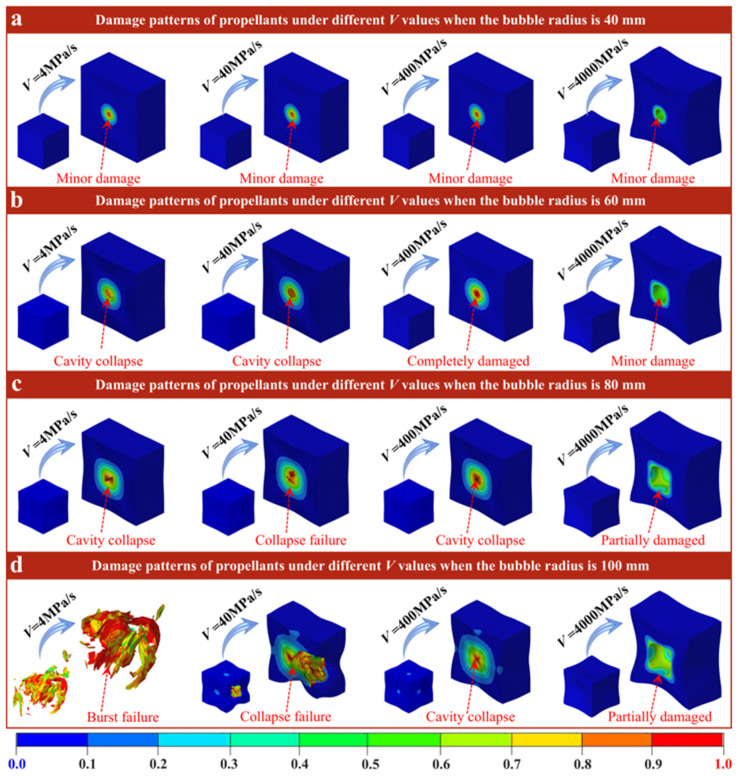
Damage nephogram of propellant with cavities of (**a**) R = 40 mm, (**b**) R = 60 mm, (**c**) R = 80 mm, (**d**) R = 100 mm at various *V*.

The final damage mode of the propellant containing four cavities’ radii at different impact rates *V* is presented in Figure 6 (where the *E* and *P* are both 16 MPa and 8 MPa). For the propellant with a cavity radius of R = 40 mm (Figure 6a), the damage is locally annular distributed, with the cavity at the center, at low- to medium-rate conditions of *V* = 4, 40 and 400 MPa/s. Moreover, the cavities become smaller, and the damage value of the inner wall is higher under confining pressure. However, the overall structure of the propellant still maintains a cube shape without obvious collapse. When *V* = 4000 MPa/s, the damage area is slightly reduced compared with the medium and low velocity, and is still confined to the region near the cavity. However, the cavities are not obviously compressed, and the damage to the cavity wall is significantly reduced. It is worth noting that the overall structure of the propellant collapses obviously at this time. For the propellant with a cavity radius of R = 60 mm (Figure 6b), the cavity cross-section is obviously crushed at *V* = 4 and 40 MPa/s. In the case of *V* = 400 MPa/s, the cross-section of the cavity remains circular, but the inner wall of the cavity also reaches the damage limit. When *V* = 400 MPa/s, the cross-section of cavities in the propellant is still round, and the damage is low. However, the collapse of the overall propellant structure is further exacerbated. When the cavity radius is R = 80 mm (Figure 6c), the cavities inside the propellant have been compressed and destroyed at the loading rate of *V* = 4 and 40 MPa/s, resulting in a slight collapse of the overall structure. At a *V* value of 400 MPa/s, the cavity section is also compressed and is close to failure. However, when *V* = 400 MPa/s, the cross-section of the cavity in the propellant becomes elliptical, and the damage to the inner wall of the cavity is aggravated. When the cavity radius is R = 100 mm (Figure 6d), the overall structure of the propellant at a velocity of 4 MPa/s is extruded and subsequently bursts. At *V* = 40 MPa/s, one side of the propellant is also crushed, but the whole structure is still retained. At *V* = 400 MPa/s, the cavities in the propellant are completely crushed, but the whole structure only collapses. At *V* = 4000 MPa/s, the cavities in the propellant still exist in an elliptical shape, but the local damage of the inner wall of the cavities is further aggravated. At the same time, the collapse behavior of the overall propellant structure is more pronounced.

On the whole, the damage degree, cavity compression characteristics, and overall structure of the propellant are regulated by *V* and R, and show a significant “Rate-dependent differential response”. At low speed, the loading time is sufficient, the load transfer is uniform, and the deformation is coordinated. On the one hand, the cavity wall can be compressed significantly in response to confining pressure, and the damage caused by stress concentration can evolve locally or globally. On the other hand, the matrix distributes the load through uniform plastic deformation, thereby avoiding local stress overload, and thus the overall structure of the propellant can maintain its cube shape without collapsing. At high speed, the loading time is very short; the load is instantaneously transferred, and the deformation cannot be coordinated. The deformation response speed of the cavity itself lags behind the load transfer speed, and it is difficult to compress; the shape remains intact. However, the confining pressure load is rapidly transferred to the propellant matrix, and the matrix is loaded by instantaneous elastic deformation, resulting in stress concentration on the four sides of the structure (a geometric weak area), which causes local overload and collapse. For small cavities, the stress concentration range is limited, and the structural collapse is slight. For medium and large cavities, the stress concentration scale increases with the radius, resulting in a more concentrated instantaneous load from the matrix, and the degree of collapse on the four sides is exacerbated. At the same time, due to the lack of damage evolution time, the damage value of the propellant is significantly reduced compared with the medium and low rates. Therefore, in practical engineering, the synergistic optimization of damage control, cavity shape preservation, and structural integrity can be achieved by adjusting the rate-dependent mechanical properties of the matrix (such as enhancing the plastic deformation ability at high rates), thereby improving the comprehensive safety of the propellant in dynamic service.

#### 5.3.3. Effect of Cavity Confining Pressure on Failure Mode

**Figure 7:**
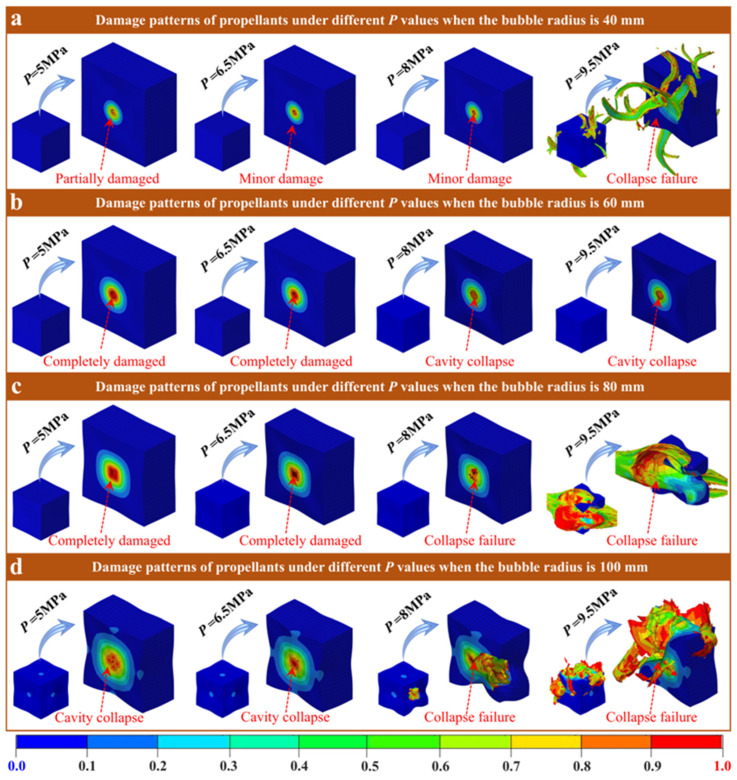
Damage nephogram of propellant containing cavities with different radius at different *P*. (**a**) R = 40 mm, (**b**) R = 60 mm, (**c**) R = 80 mm, (**d**) R = 100 mm.

Figure 7 presents the final damage mode of the propellant containing four different cavity radii under varying cavity confining pressures *P* (at this time, both *E* and *V* are 16 MPa and 40 MPa/s, respectively). For the propellant with a cavity radius of R = 40 mm (Figure 7a), the cavity cross-section within the propellant remains circular, and the stress concentration is confined to the region near the cavity wall at *P* = 5, 6.5, and 8 MPa conditions. Moreover, as the increase in confining pressure, the damage to the inner wall of the cavity increases gradually. When *P* rises to 9.5 MPa, the local stress overload causes the cavity center collapses. However, due to the small cavity size, the stress concentration only leads to the extrusion damage on the surface of the propellant structure, and no global rupture failure occurs. For the propellant with a cavity radius of R = 60 mm (Figure 7b), the cavity section remains circular at *P* = 5 and 6.5 MPa conditions, but the damage is close to the limit. At *P* = 8 and 9.5 MPa, the cavity has been completely squeezed and collapsed. However, overall, the cavity shape and the overall structure of the propellant remain stable without obvious damage characteristics, reflecting the propellant’s good tolerance to confining pressure under the cavity radius. At a cavity radius of R = 80 mm (Figure 7c), the cavity cross-section within the propellant transforms to a square at *P* = 5 and 6.5 MPa, and the damage area expands further. At a pressure of 8 MPa, the cavities are completely crushed, and the surrounding matrix is destroyed; however, the overall structure of the propellant remains intact. At a pressure of 9.5 MPa, the cavity breaks, and the entire propellant structure exhibits burst failure. When the cavity radius is R = 100 mm (Figure 7d), the cavity was completely squeezed and collapsed at pressures of *P* = 5 and 6.5 MPa, and the damage extended from the cavity area to the periphery, deforming the propellant structure. However, at pressures of *P* = 8 and 9.5 MPa, the cavities break, and the overall structure of the propellant is destroyed. Especially at *P* = 9.5 MPa, the entire structure of the propellant is severely distorted, and the cube shape is completely lost, exhibiting complete failure characteristics.

From the above results, it can be observed that the failure mode and structural stability of the propellant are jointly regulated by *P* and cavity radius, exhibiting a cavity size-dependent characteristics in failure. For a small cavity of R = 40 mm, the stress concentration area is very small, and the load is carried by the local elastic deformation around the cavity under low confining pressure. When the confining pressure exceeds the critical value, local stress overload causes the cavity center to collapse. However, the stress does not exceed the loading threshold of the whole matrix, so the overall structure of the propellant retains its the cubic shape. For medium cavities of R = 60 mm, the range of stress concentration is compatible with the matrix’s plastic deformation buffer zone. Even under high confining pressure, the load can be dispersed by local plastic deformation, thus avoiding the occurrence of extrusion/compression failure. But for large cavities of R = 80 and 100 mm, the range of stress concentration far exceeds the cushioning capacity of local deformation. Under low confining pressure, the stress diffuses throughout the entire structure, and with the increase in confining pressure, the stress overload effect intensifies. Finally, the failure of the propellant evolves from compression failure in the cavity region to morphological distortion failure of the overall structure. Therefore, it is recommended that a cavity radius of R = 60 mm be used as the target control size in practical engineering, and the corresponding confining pressure conditions should be matched accordingly. At the same time, the local bearing capacity of small cavity areas should be strengthened, and the propellant’s confining pressure service safety can be improved from two aspects: cavity control and performance optimization.

## 6. Discussion

Based on the analysis of the system parameters described above, the failure of solid propellants under dynamic loading is governed by competing physical mechanisms. Specifically, the final failure mode is controlled by three coupled factors: stress concentration governed by defect size, inertial effects under high-rate loading, and the combined stiffness of the material and external confinement. Large cavities (R = 100 mm) constitute pronounced geometric discontinuities and generate substantial stress concentrations during the early loading stage. Under quasi-static or moderate loading rates (e.g., *V* = 40 MPa/s), stress concentration dominates, and damage initiates and propagates rapidly along the inner cavity wall. This process culminates in cavity crushing and a cloverleaf-like failure pattern (Figure 4c,d). In contrast, at extremely high loading rates (*V* = 4000 MPa/s), inertial effects become dominant. Inertial effects slow the propagation of localized cracks and, more importantly, redirect the energy-dissipation pathway. Simulations indicate that, although localized damage is reduced, the input kinetic energy is redistributed into global structural deformation. Consequently, global collapse replaces localized perforation as the primary failure mode (Figure 6). These results explain why, at ultra-high strain rates, global structural stability becomes the primary safety-limiting factor. In addition, the propellant’s *E* and *P* act as key boundary conditions that regulate the competing mechanisms described above. A higher modulus (*E* ≥ 24 MPa) increases material stiffness and thereby confines the damage zone. Even for larger defects (R = 80 mm), the increased stiffness limits damage to a localized annular region and prevents global structural failure (Figure 5). In contrast, the external confining pressure provides a direct driving force for collapse. As P increases, the hydrostatic pressure on the cavity wall eventually exceeds the material strength, triggering cavity collapse. The critical pressure threshold decreases with increasing cavity size (Figure 7). These results indicate that defect stability depends on both internal factors (material stiffness) and external conditions (confinement/loading).

The competing mechanisms described above provide a clear physical framework and a rational basis for engineering decisions. In solid rocket motor development, defect-tolerance criteria and material properties (e.g., *E*) should be defined jointly with the mission load spectrum, including *V* and *P*. For motors experiencing high overload, design should prioritize resistance to global structural collapse, and local strength requirements for small defects can be relaxed where justified. In contrast, under sustained high internal pressure, large defects must be tightly controlled, and the material modulus must be sufficient to prevent pressure-induced defect instability. The quantitative parameter–failure-mode correlations and the simulation workflow developed in this study enable integrated performance–defect–load co-design, supporting a shift in solid propulsion systems from empirical safety margins toward predictive safety.

## 7. Conclusions

In this study, a constitutive model for solid propellant was established based on the GISSMO model, and its validity was verified through uniaxial tensile tests conducted at various tensile rates. Building on this validated model, the failure behavior of solid propellants with different cavity sizes under dynamic compression loading was simulated. The main conclusions are as follows.

The study first validated the GISSMO model’s ability to predict propellant rate-dependent mechanical responses, with errors below 10% and accurate capture of the transition from elastic–plastic to hyperelastic behavior. Building on this foundation, simulation results show that cavity size governs damage patterns. Large cavities (R ≥ 80 mm) induce localized collapse or cross-shaped tearing due to significant stress concentration, while small cavities (R ≤ 60 mm) maintain stability. The initial modulus further influences damage propagation. When the initial modulus exceeds 24 MPa, it suppresses global damage caused by large cavities. Impact velocity governs energy dissipation pathways. At lower velocities (*V* ≤ 400 MPa/s), loads are absorbed through cavity compression, while at high velocities (*V* = 4000 MPa/s), inertial effects transfer energy to the overall structure, causing matrix collapse. Moreover, the failure threshold of cavity confining pressure is related to cavity size. Smaller cavities withstand higher confining pressures without instability, while larger cavities may undergo crushing failure over a broader pressure range.

This study developed a refined model of solid propellants containing cavity defects and, in conjunction with the GISSMO constitutive model, explored the influence of cavity size on the material’s dynamic failure behavior under fixed external constraints. Future research should focus on constructing scaled specimen models with a fixed void volume fraction to decouple the material’s intrinsic mesoscopic size effects from external structural boundary effects, allowing for a more comprehensive understanding of the underlying mechanisms associated with cavities. Additionally, incorporating an appropriate gas equation of state and using fluid–structure interaction methods are essential to quantify the dynamic behavior of gas within cavities under impact loading and its influence on damage evolution. Overall, this study offers valuable simulation-based insights into assessing the structural integrity of solid propellants.

## Data Availability

Data will be made available on request.
